# Interaction Effects of Maternal Sexually Transmitted Infections with Prenatal Care Utilization Status on Preterm Birth and Low Birthweight: U.S. National Data

**DOI:** 10.3390/jcm11175184

**Published:** 2022-09-01

**Authors:** Anthony J. Kondracki, Wei Li, Zoran Bursac, Manouchehr Mokhtari, Bonzo Reddick, Jennifer L. Barkin

**Affiliations:** 1 Department of Community Medicine, Mercer University School of Medicine, Macon, GA 31207, USA; 2 Department of Psychiatry, Yale University School of Medicine, New Haven, CT 06510, USA; 3 Department of Biostatistics, Robert Stempel College of Public Health & Social Work, Florida International University, Miami, FL 33199, USA; 4 Department of Family Science, University of Maryland School of Public Health, College Park, MD 20742, USA

**Keywords:** interaction analysis, sexually transmitted infections, prenatal care, preterm birth, low birthweight

## Abstract

This case-control study aimed to test interaction between the most common sexually transmitted infections (STIs) (i.e., chlamydia, gonorrhea, and syphilis) and prenatal care (PNC) utilization status on preterm birth (PTB) (<37 weeks gestation) and low birthweight (LBW) (<2500 g). We used data of participants with singleton live births (N = 3,418,028) from the 2019 United States National Vital Statistics System. There were 280,206 participants in the PTB group and 3,137,822 in the control group, and 221,260 participants in the LBW group and 3,196,768 in the control group. Nearly 1.9% of the participants had chlamydia, 0.3% had gonorrhea, and 0.2% had syphilis. Interaction effects of STIs with PNC utilization status on the risk of PTB and LBW were tested on the multiplicative and additive scales. Using measures of the relative excess risk of interaction (RERI), the attributable proportion of interaction (AP), and the synergy index (S), we observed the highest significant synergistic interaction between syphilis and inadequate PNC utilization increasing the risk of PTB (RERI 2.12, AP 38%, and SI 1.88), and between gonorrhea and inadequate PNC utilization increasing the risk of LBW (RERI 1.03, AP 28%, and SI 1.64). Findings from this study help improve our understanding of disease etiology and inform prevention planning.

## 1. Introduction

It is important to address adverse newborn outcomes in the context of the epidemic of sexually transmitted infections (STIs). Recent national surveillance data show a sustained rise in STI rates in the general population of the United States, reaching an all-time high for the sixth consecutive year [1]. In 2019, there were 1,808,703 reported cases of *Chlamydia trachomatis* (chlamydia) infection (19% increase since 2015), 616,392 cases of *Neisseria gonorrhoeae* (gonorrhea) (56% increase since 2015), and nearly 130,000 cases of primary and secondary *Treponema pallidum* (syphilis) infection (74% increase since 2015) [1]. In 2014, the U.S. Preventive Services Task Force (USPSTF) reissued recommendations for screening for chlamydia and gonorrhea in sexually active women between 24 years old or younger and in older women at increased risk for infection [2]. If acquired before or during pregnancy and not treated, some STIs (e.g., syphilis) can cross the placental barrier causing premature rupture of membranes (PROM), stillbirth, congenital syphilis, preterm birth, and low birthweight [1,2,3,4,5]. In the U.S. from 2016 to 2018, chlamydia rates among pregnant women increased 2%, gonorrhea rates increased 16%, and syphilis rates increased 34% [6]. During the same time, the prevalence of preterm birth (PTB) (<37 weeks gestation) and low birthweight (LBW) (<2500 g) also increased. In 2019, the rate of PTB was 10.23% (up from 10.02% in 2018) and the overall rate of LBW was 8.31% [7]. A recent study reported increased PTB rates in association with maternal STIs using birth certificate data from the U.S. National Vital Statistics System (NVSS) [8]. Pregnant women can obtain screening, treatment, and prevention counselling for STIs during their routine prenatal care (PNC) visits. The American College of Obstetricians and Gynecologists (ACOG) and the American Academy of Pediatrics (AAP) recommend initiating PNC early in the first trimester of pregnancy and receiving care every 4 weeks for the first 28 weeks, then every 2 to 3 weeks until 36 weeks of pregnancy, and weekly until delivery [9]. In 2019, 76.7% of pregnant women received early and adequate PNC, but the target of 80.5% set by Healthy People 2030 may be difficult to achieve [10]. Prior studies have demonstrated the association between numerous risk factors and the benefits of PNC in improving pregnancy outcomes [11,12,13]. However, synergistic interaction between maternal STIs and PNC utilization status on the risk of PTB and LBW have not been commonly reported. This case-control study using data from the U.S. National Vital Statistics System was conducted to investigate whether the combined effect of STIs and PNC utilization on the risk of PTB and LBW is a simple sum of their independent effects, or whether they act synergistically and the effect is greater than the sum of their independent effects. Given the complexity of PTB and LBW [14,15], testing interaction effects can improve our understanding of the relationship between the risk factors underpinning disease etiology and prevention. For a transparent presentation, interaction was assessed on both multiplicative and additive scales [16,17].

## 2. Materials and Methods

### 2.1. Data Source and Study Population

This case-control study used data from the United States National Center for Health Statistics (NCHS), National Vital Statistics System (N = 3,757,582), and based on the information obtained from the 2019 standard certificate of live birth [8]. The 2003 revision of a birth certificate implemented in 2016 in all 50 states and the District of Columbia contains information on the number of PNC visits, the trimester in which care began, the Adequacy of Prenatal Care Utilization (APNCU) index [18], and “infections present and/or treated during this pregnancy [6,19]”. Data were restricted to participants with singleton births because of a different risk profile in multiple gestations. Multiple births (*n* = 123,995) and missing observations for any covariates were deleted and complete case data of participants (*n* = 3,418,028) were used in this study analyses. Participants were divided into a PTB group (*n* = 280,206) and a control group (*n* = 3,137,822) and an LBW group (*n* = 221,260) and a control group (*n* = 3,196,768).

### 2.2. Exposure and Outcome Ascertainment

Maternal STIs (risk factors) and PNC utilization status (a preventive factor) were exposure variables. The STIs of interest were chlamydia, gonorrhea, and syphilis, categorized as present infection or no infection, and PNC utilization status was based on the Adequacy of the Prenatal Care Utilization (APNCU) index [20] and categorized as adequate, if PNC began early in the first trimester and included more than 10 visits, or as inadequate, if PNC began in the second/third trimester and included less than 10 visits. Two potential outcomes were PTB (less than 37 weeks gestation) and LBW (less than 2500 g) [19]. Gestational age was assessed using obstetric estimate that combines the date of the last menstrual period and ultrasound measurement for validity [21].

### 2.3. Covariate Assessment

Covariates were chosen based on prior knowledge [11,12,13,14,15] and availability in the dataset. Race (i.e., non-Hispanic White, non-Hispanic Black, non-Hispanic Asian, non-Hispanic American Indian/Alaska Native, or mixed races) and Hispanic ethnicity were defined consistent with the Office of Management and Budget standards [22]. Age categories were less than 20, 20–24, 25–29, 30–34, 35 years or more, and education categories were high school or less, some college, and a bachelor’s degree or higher. Health insurance categories included Medicaid/other public, private, or self-pay/uninsured. Marital status was classified as married or unmarried, and parity was classified as primipara who had one previous birth and multipara who had more than one birth. Additional dichotomous covariates were history of previous preterm birth, gestational diabetes, gestational hypertension, self-reported smoking, and infant sex (male or female).

### 2.4. Statistical Analyses

The prevalence rate of PTB and LBW and distribution of chlamydia, gonorrhea, and syphilis were examined in association with race/ethnicity, age, education, marital status, health insurance, liveborn parity, prior preterm birth, gestational diabetes, gestational hypertension, prepregnancy body mass index (BMI), PNC utilization status, smoking, STIs, alcohol use, drug use, depression, and infant sex. The main effects of STIs and PNC status in association with PTB and LBW were assessed in two separate multivariable logistic regression models assessed and adjusted for relevant covariates. Next, four mutually exclusive interaction term categories were created from arbitrarily dichotomized STIs (Factor 1) and PNC status (Factor 2) [i.e., exposure to Factor 1 and not to Factor 2 (+−); no exposure to Factor 1 and exposure to Factor 2 (−+); exposure to both Factors (joint exposure) (++); and no exposure to any Factor (−−)] and tested in logistic regression models, adjusting for covariates. Assessment was conducted on the multiplicative scale and on the additive scale that included measures of the relative excess risk due to interaction (RERI), the attributable proportion (AP), and the synergy index (SI) [23,24]. To prevent errors in estimates when both factors were considered jointly, the PNC variable (a preventive factor) was recoded to a risk factor and an interaction subgroup with the lowest risk for PTB and LBW was used as a reference category [25]. As a sensitivity analysis, because the distribution of STIs among pregnant women differed across age groups, we further conducted a stratified analysis by age < 25 years old and ≥25 years old) replicating all of the above multivariate analyses for interaction. Tables with inconsistent interaction estimates before the PNC variable was recoded, were also presented for comparison. SAS software version 9.4 (SAS Institute Inc., Cary, NC, USA) was used in all statistical analyses. The significance level was set at α = 0.05 in conjunction with the rest of the evidence on measures of the association, risk, and variability described below. This was a secondary analysis of deidentified and publicly available data, and the study did not require approval by the Institutional Review Board.

### 2.5. Interaction Measures

Interaction in epidemiological research denotes a relationship (or effect) of one risk factor with an outcome that differs across strata of another risk factor [23]. Testing the presence of interaction on a multiplicative and an additive scale requires an interaction term added to the regression model. Multiplicative interaction means that the combined effect of exposure to two risk factors is larger (or smaller) than the product of their individual effects, and an additive interaction means that the combined effect from two exposures is larger (or smaller) than the sum of their individual effects [23,26,27]. Multiplicative interaction was calculated using the formula: RR_11_/(RR_10_ × RR_01_), where RR_11_ is the relative risk from exposure to both risk factors; RR_10_ is the relative risk from exposure to the first risk factor; and RR_01_ is the relative risk of exposure to the second risk factor [24,26]. Additive interaction measures included the relative excess risk due to interaction (RERI), the attributable proportion (AP), the synergy index (SI), and their 95% confidence intervals (CIs) based on the delta method [28].

The RERI was calculated using the formula: [RR_11_ − RR_10_ − RR_01_ + 1] and interpreted as the excess risk due to exposure to both risk factors relative to the risk without exposure to one or the other factor. The AP was calculated using the formula: [RERI/RR_11_] and interpreted as the proportion of excess disease risk due to interaction between exposures to both risk factors [29]. The SI was assessed using the formula: [(RR_11_ − 1)]/ [(RR_10_ − 1) + (RR_01_ − 1)] and interpreted as the excess risk due to exposure to both risk factors with interaction, relative to the excess risk due to exposure to both risk factors without interaction [24,26]. The interaction is interpreted as superadditive when RERI > 0, AP > 0, and SI > 1 or sub-additive when RERI < 0, AP < 0, and SI < 1. There is no interaction if the CI of RERI and AP contains 0 and the CI of S contains 1.

Although interaction effects are generally examined using the risk ratios (RRs), the odds ratios (ORs) will approximate RRs when the outcome is rare (<10%) [29]. However, prior studies reported discrepancies in multiplicative interaction [30] and in RERI estimates [31,32] when using odds ratios (ORs). In our study, PNC, a preventive factor, had to be recoded to a risk factor to avoid error in interaction estimates [25]. It has been argued that interaction tested on the additive scale has greater statistical power to assess “biologic interaction” by detecting differences in effect measures [23,26,33] than multiplicative interaction which captures a proportion effect between risk factors.

## 3. Results

### 3.1. Descriptive Analyses

There were 280,206 (8.2%) participants in the PTB group and 3,137,822 (91.8%) in the control group, and 221,260 (6.5%) participants in the LBW group and 3,196,768 (93.5%) in the control group (Table 1). PTB and LBW were most prevalent among non-Hispanic Black women, younger (<20 years old) or older (≥35 years old) women, with a high school education or less, unmarried, multiparas, and on Medicaid. As indicated in Table 2, 64,443 (1.9%) participants reported chlamydia, 10,913 (0.3%) reported gonorrhea, and 4984 (0.2%) reported syphilis, which all three infections were most common among non-Hispanic Black women, less educated (a high school education or less), women on Medicaid, and in multiparas.

### 3.2. Main Effects on PTB and LBW

The main effects of STIs and PNC utilization in the association with PTB and LBW were estimated under the assumption of no interaction (Table 3, Model 1 and Model 2). The odds ratios of PTB in association with chlamydia were 2% higher (aOR 1.02, 95% CI: 1.01, 1.04), 13% higher (aOR 1.13, 95% CI: 1.07, 1.20) in association with gonorrhea, and 39% higher (aOR 1.39, 95% CI: 1.28, 1.51) in association with syphilis, compared to no infection. Likewise, the odds ratios of LBW in association with chlamydia were 4% higher (aOR 1.04, 95% CI: 1.00, 1.06), 14% higher (aOR 1.14, 1.07, 1.21) in association with gonorrhea, and 34% higher (aOR 1.34, 95% CI: 1.22, 1.46) in association with syphilis, relative to no infection. Moreover, the odds ratios of PTB were 64% lower (aOR 0.36, 95% CI: 0.35, 0.36) and the odds ratios of LBW were 59% lower (aOR 0.41, 95% CI: 0.40, 0.41) in association with adequate PNC utilization, compared with inadequate PNC utilization.

### 3.3. Interaction Effects of STIs with Inadequate PNC Utilization on the Risk of PTB

In Table 4, the odds ratios of PTB were over 3.5-fold higher (aOR 3.56, 95% CI: 3.15, 4.02; *p* < 0.001) due to chlamydia and inadequate PNC utilization, were over 5-fold higher due to gonorrhea and inadequate PNC (aOR 5.06, 95% CI: 4.10, 6.25; *p* < 0.001), and over 5.5-fold higher (aOR 5.54, 95% CI: 4.24, 7.24; *p* < 0.001) due to syphilis and inadequate PNC, relative to no infection and adequate PNC (baseline risk category). The synergistic interaction on the overall increased risk of PTB was the strongest between syphilis and PNC utilization. The multiplicative interaction generally reached statistical significance and the interaction was superadditive (RERI 2.12, AP 38%, and SI 1.88) and statistically significant.

### 3.4. Interaction Effects of STIs with Inadequate PNC Utilization on the Risk of LBW

In Table 5, the odds ratios of LBW were almost 3-fold higher (aOR 2.76, 95% CI: 2.42, 3.16; *p* < 0.001) due to chlamydia and inadequate PNC, over 3.6-fold higher (aOR 3.64, 95% CI: 2.92, 4.55; *p* < 0.001) due to gonorrhea and inadequate PNC, and over 3-fold higher (aOR 3.22, 95% CI: 2.39, 4.35; *p* < 0.001) due to syphilis and inadequate PNC, relative to no infection and adequate PNC utilization. The observed synergistic interaction increasing the risk of LBW was the strongest between gonorrhea with PNC utilization. The multiplicative interaction measure mostly reached statistical significance and the interaction was superadditive (RERI 1.03, AP 28%, and SI 1.64) and statistically significant across each interaction subgroup.

### 3.5. Interaction Effects Stratified by Age (<25 and ≥25 Years Old)

There were 67,803 participants in the PTB group and 59,985 in the LBW group who were less than 25 years old, and 212,403 participants in the PTB group and 161,275 in the LBW group who were 25 years old or older (Supplementary Tables S1–S4). Among participants < 25 years old (Tables S1 and S3, Supplementary Information), the strongest significant synergistic interaction was observed between syphilis and inadequate PNC for both the risk of PTB (aOR 4.36, 95% CI: 2.66, 7.15; *p* < 0.001) and LBW (aOR 3.43, 95% CI: 2.02, 5.82; *p* < 0.001). Additive interaction estimates were less stable, possibly due to a small sample size, however, the synergy index (SI) was 1.38 on PTB and 1.40 on LBW. By comparison, among participants ≥ 25 years old (Supplementary Tables S2 and S4), the strongest significant synergistic interaction was observed between gonorrhea and inadequate PNC for both the odds of PTB (aOR 6.15, 95% CI: 4.66, 8.10; *p* < 0.001) and LBW (aOR 4.09, 95% CI: 3.07, 5.47; *p* < 0.001). Now, the multiplicative interaction reached statistical significance and interaction was superadditive and statistically significant. Supplementary Tables S5 and S6 show inconsistent estimates across subgroups of interaction before the PNC variable was recoded.

## 4. Discussion

In spite of screening recommendations, common and treatable bacterial STIs continue to be a major and growing public health challenge, particularly when affecting pregnancies. We were interested in identifying subpopulations of pregnant women with STIs and inadequate PNC who could be targeted in interventions to lower the risk of PTB and LBW. In our study, chlamydia was the most commonly reported STI, and syphilis was the least commonly reported. Chlamydia and gonorrhea occurred mostly among women 20–24 years old and syphilis among women 25–29 years old. All three STIs were associated with significantly increased risk of PTB and LBW, however, syphilis had the strongest association increasing the risk of both PTB and LBW over 30%. It has been previously established that adequate PNC utilization can lower the risk of adverse pregnancy outcomes [11,12,13]. In a recent national report, women of younger age, with a lower level of education, and having a fourth or higher birth order were less likely to begin care in the first trimester and were more likely to receive late or no PNC [18]. In our study, adequate PNC utilization accounted for 64% lower odds of PTB and 59% lower odds of LBW, compared with inadequate PNC. Interaction effects between the risk factors differed by the participants’ age. In women < 25 years old, syphilis and inadequate PNC utilization had the strongest synergistic effect on both PTB (SI 1.38, AP 21%) and LBW (SI 1.40, AP 20%). However, in women ≥ 25 years old, interaction between gonorrhea and inadequate PNC utilization had the strongest effect on both PTB (SI 2.34, AP 48%) and LBW (SI 1.97, AP 37%). These findings suggest that younger women (<25 years old) with syphilis and older women (≥25 years old) with gonorrhea are most likely to benefit from adequate PNC interventions to avert PTB and LBW, which is considered clinically relevant and of public health significance. It should also be mentioned that the odds of PTB and LBW were about 3-fold higher due to interaction between no chlamydia, no gonorrhea, and no syphilis infection and inadequate PNC utilization, compared with no infection and adequate PNC. This indicates that in women with inadequate PNC, other risk factors can explain the association with adverse newborn outcomes. The multifactorial etiology of PTB and LBW involves many risk factors and shared pathophysiological mechanisms influencing fetal development and in the course of pregnancy [14,15]. While most STIs entering the uterus are asymptomatic, they account for about 40% of PTBs [34]. PTB and LBW are leading causes of neonatal death and under-5 infant mortality, as well as morbidity and disability later in life [15]. Reproductive epidemiology has a well-justified concern regarding timing of screening and eliminating any potentially harmful exposures occurring during a gestational vulnerability window [35] to allow for a healthy progress of pregnancy [11,12,13]. Women infected with gonorrhea in pre-conception or in the first trimester of pregnancy, rather than later in pregnancy, are at a greater risk for PTB [36]. Using interaction analysis in assessing risk differences for multifactorial diseases can benefit health promotion and disease prevention interventions where eliminating some risk factors under the synergistic assumption is essential [33].

There are strengths and limitations of this study. To the best of our knowledge, this is the first study based on national data exploring the potential synergistic interaction effects of maternal STIs (risk factors) with inadequate PNC utilization (a preventive factor) on PTB and LBW. Availability of a large dataset of over 3 million participants provided cases and controls, as well as many covariates and sufficient statistical power to explore interaction on the multiplicative and additive scales. The first limitation is that causal relationships could not be inferred because the exposure was not randomized and, despite adjustments, estimates were subject to potential confounding. Second, recall or selection bias from self-reports may have resulted in misclassification. Third, there was a lack of information in the dataset on timing of diagnosis and treatment of STIs of interest in our study and on other coinciding STIs (e.g., bacterial vaginosis, trichomonas) [35,37]. Moreover, no information was available on specific subtypes of PTB (e.g., spontaneous, medically induced, preterm premature rupture of membranes) [15] to allow for outcome assessment.

## 5. Conclusions

This study highlighted significant synergistic interaction effects of common and treatable STIs with inadequate PNC utilization increasing the risk of PTB and LBW to improve our understanding of disease etiology and inform prevention planning. A contribution to the existing literature is demonstrating methodologically how interaction analysis may be useful in assessing risk differences in observational and experimental research.

## Figures and Tables

**Table 1:** Characteristics of participants with singleton preterm birth and low birthweight (Total *n* = 3,418,028).

Variables	Preterm Birth Groups			Low Birthweight Groups		
	Case *n* = 280,206 (8.2%)	Control *n* = 3,137,822 (91.8%)	*p* Value	Case *n* = 221,260 (6.5%)	Control *n* = 3,196,768 (93.5%)	*p* Value
***Race/ethnicity***			<0.001			<0.001
Non-Hispanic White	127,646 (7.2)	1,644,164 (92.8)		90,426 (5.1)	1,681,384 (94.9)	
Non-Hispanic Black	57,927 (11.8)	434,420 (88.2)		57,152 (11.6)	435,195 (88.4)	
Hispanic	68,741 (8.4)	753,197 (91.6)		50,204 (6.1)	771,734 (93.9)	
Other races ^a^	25,892 (7.8)	306,041 (92.2)		23,478 (7.1)	308,455 (92.9)	
***Age, years***			<0.001			<0.001
<20	14,541 (9.1)	145,921 (90.9)		14,046 (8.7)	146,416 (91.3)	
20–24	53,262 (8.2)	595,299 (91.8)		45,939 (7.1)	602,622 (92.9)	
25–29	75,495 (7.6)	912,237 (92.4)		59,506 (6.0)	928,226 (94.0)	
30–34	76,026 (7.7)	915,057 (92.3)		57,492 (5.8)	933,591 (94.2)	
≥35	60,882 (9.7)	569,308 (90.3)		44,277 (7.0)	585,913 (93.0)	
***Education level***			<0.001			<0.001
High school or less	123,482 (9.4)	1,186,621 (90.6)		103,094 (7.9)	1,207,009 (92.1)	
Some college	83,265 (8.7)	877,047 (91.3)		63,442 (6.6)	896,870 (93.4)	
Bachelor’s or higher	73,459 (6.4)	1,074,154 (93.6)		54,724 (4.8)	1,092,889 (95.2)	
***Marital status***			<0.001			<0.001
Married	130,352 (7.2)	1,677,786 (92.8)		93,512 (5.2)	1,714,626 (94.8)	
Unmarried	149,854 (9.3)	1,460,036 (90.7)		127,748 (7.9)	1,482,142 (92.1)	
***Health insurance***			<0.001			<0.001
Medicaid/other public	145,937 (9.4)	1,405,608 (90.6)		122,152 (7.9)	1,429,393 (92.1)	
Private	123,658 (7.2)	1,595,051 (92.8)		91,095 (5.3)	1,627,614 (94.7)	
Self-pay/none	10,611 (7.2)	137,163 (92.8)		8013 (5.4)	139,761 (94.6)	
***Parity***			<0.001			<0.001
Primipara	109,508 (8.2)	1,217,530 (91.8)		100,775 (7.6)	1,226,263 (92.4)	
Multipara	170,698 (16.4)	1,920,292 (83.6)		120,485 (11.5)	1,970,505 (88.5)	
***Prior preterm birth***	32,381 (26.8)	88,569 (73.2)	<0.001	21,145 (17.5)	99,805 (82.5)	<0.001
***Gestational diabetes***	26,960 (11.5)	207,106 (88.5)	<0.001	16,252 (6.9)	217,814 (93.1)	<0.001
***Gestational hypertension***	48,880 (18.9)	209,848 (81.1)	<0.001	39,329 (15.2)	219,399 (84.8)	<0.001
***Smoking***	24,751 (12.2)	178,666 (87.8)	<0.001	24,930 (12.3)	178,487 (87.7)	<0.001
***STIs***			<0.001			<0.001
Chlamydia	6477 (10.0)	57,966 (90.0)		5989 (9.3)	58,454 (90.7)	
Gonorrhea	1348 (12.3)	9565 (87.7)		1283 (11.8)	9630 (88.2)	
Syphilis	729 (14.6)	4255 (85.4)		636 (12.8)	4348 (87.2)	
***PNC utilization status ^b^***			<0.001			<0.001
PNC adequate	212,556 (8.0)	2,440,860 (92.0)		161,986 (6.1)	2,491,430 (93.9)	
PNC inadequate	67,650 (8.8)	696,962 (91.2)		59,274 (7.7)	705,338 (92.3)	
***Infant sex***			<0.001			<0.001
Male	150,375 (8.6)	1,598,990 (91.4)		103,388 (5.9)	1,645,977 (94.1)	
Female	129,831 (7.8)	1,538,832 (92.2)		117,872 (7.1)	1,550,791 (92.9)	

^a^ Other races: Asian, American Indian/Alaska Native, Native Hawaiian, Pacific Islander or mixed races; STIs: sexually transmitted infections; ^b^ PNC: prenatal care; PNC adequate: early and >10 visits; PNC inadequate: late and ≤10 visits.

**Table 2:** Distribution of chlamydia, gonorrhea, and syphilis infections in the study sample.

Variables	Total *n* = 3,418,028 (100%)	Chlamydia *n* = 64,443 (1.9%)	No Chlamydia *n* = 3,353,585 (98.1%)	*p* Value	Gonorrhea *n* = 10,913 (0.3%)	No Gonorrhea *n* = 3,407,115 (99.7%)	*p* Value	Syphilis *n* = 4,984 (0.2%)	No Syphilis *n* = 3,413,044 (99.8%)	*p* Value
***Race/ethnicity***				<0.001			<0.001			<0.001
Non-Hispanic White	1,771,810 (51.8)	20,006 (31.0)	1,751,804 (52.2)		3151 (28.9)	1,768,659 (51.9)		1214 (24.4)	1,770,596 (51.9)	
Non-Hispanic Black	492,347 (14.4)	21,109 (32.8)	471,238 (14.1)		5007 (45.9)	487,340 (14.3)		1986 (39.9)	490,361 (14.4)	
Hispanic	821,938 (24.1)	18,373 (28.5)	803,565 (24.0)		1815 (16.6)	820,123 (24.1)		1383 (27.7)	820,555 (24.0)	
Other races ^a^	331,933 (9.7)	4955 (7.7)	326,978 (9.7)		940 (8.6)	330,993 (9.7)		401 (8.0)	331,532 (9.7)	
***Age, years***				<0.001			<0.001			<0.001
<20	160,462 (4.7)	12,210 (18.9)	148,252 (4.4)		1861 (17.0)	158,601 (4.6)		330 (6.6)	160,132 (4.7)	
20–24	648,561 (19.0)	27,006 (41.9)	621,555 (18.5)		4182 (38.3)	644,379 (18.9)		1258 (25.2)	647,303 (19.0)	
25–29	987,732 (28.9)	15,725 (24.4)	972,007 (29.0)		2900 (26.6)	984,832 (28.9)		1528 (30.7)	986,204 (28.9)	
30–34	991,083 (29.0)	6741 (10.5)	984,342 (29.4)		1429 (13.1)	989,654 (29.1)		1133 (22.7)	989,950 (29.0)	
≥35	630,190 (18.4)	2761 (4.3)	627,429 (18.7)		541 (5.0)	629,649 (18.5)		735 (14.8)	629,455 (18.4)	
***Education***				<0.001			<0.001			<0.001
High school or less	1,310,103 (38.3)	45,076 (69.9)	1,265,027 (37.7)		8029 (73.6)	1,302,074 (38.2)		3388 (68.0)	1,306,715 (38.3)	
Some college	960,312 (28.1)	15,837 (24.6)	944,475 (28.2)		2416 (22.1)	957,896 (28.1)		1211 (24.3)	959,101 (28.1)	
Bachelor’s or higher	1,147,613 (33.6)	3530 (5.5)	1,144,083 (34.1)		468 (4.3)	1,147,145 (33.7)		385 (7.7)	1,147,228 (33.6)	
***Marital status***				<0.001			<0.001			<0.001
Married	1,808,138 (52.9)	9975 (15.5)	1,798,163 (53.6)		1207 (11.1)	1,806,931 (53.0)		991 (19.9)	1,807,147 (53.0)	
Unmarried	1,609,890 (47.1)	54,468 (84.5)	1,555,422 (46.4)		9706 (88.9)	1,600,184 (47.0)		3993 (80.1)	1,605,897 (47.0)	
***Health insurance***				<0.001			<0.001			<0.001
Medicaid/other public	1,551,545 (45.4)	49,945 (77.5)	1,501,600 (44.8)		9080 (83.2)	1,542,465 (45.3)		3951 (79.2)	1,547,594 (45.4)	
Private	1,718,709 (50.3)	12,225 (19.0)	1,706,484 (50.9)		1577 (14.5)	1,717,132 (50.4)		834 (16.8)	1,717,875 (50.3)	
Self-pay/None	147,774 (4.3)	2273 (3.5)	145,501 (4.3)		256 (2.3)	147,518 (4.3)		199 (4.0)	147,575 (4.3)	
***Parity***				<0.001			<0.001			<0.001
Primipara	1,327,038 (38.8)	30,858 (47.9)	1,296,180 (38.7)		4222 (38.7)	1,322,816 (38.8)		1549 (31.1)	1,325,489 (38.8)	
Multipara	2,090,990 (61.2)	33,585 (52.1)	2,057,405 (61.3)		6691 (61.3)	2,084,299 (61.2)		3435 (68.9)	2,087,555 (61.2)	
***Prior preterm birth***	120,950 (3.5)	3056 (4.7)	117,894 (3.5)	<0.001	700 (6.4)	120,250 (3.5)	<0.001	358 (7.2)	120,592 (3.5)	<0.001
***Gestational diabetes***	234,066 (6.8)	3223 (5.0)	230,843 (6.9)	<0.001	487 (4.5)	233,579 (6.8)	<0.001	347 (7.0)	233,719 (6.8)	0.747
***Gestational hypertension***	258,728 (7.6)	6075 (9.4)	252,653 (7.5)	<0.001	977 (9.0)	257,751 (7.6)	<0.001	529 (10.6)	258,199 (7.6)	<0.001
***Smoking***	203,417 (5.9)	8122 (12.6)	195,425 (5.8)	<0.001	2293 (21.0)	201,124 (5.9)	<0.001	758 (15.2)	202,659 (5.9)	<0.001
***PNC utilization status ^b^***				<0.001			<0.001			<0.001
PNC adequate	1,892,747 (55.4)	26,836 (41.6)	1,865,911 (55.6)		4066 (37.2)	1,888,681 (55.4)		1911 (38.3)	1,890,836 (55.4)	
PNC inadequate	764,612 (44.6)	23,535 (58.4)	741,077 (44.4)		4276 (62.8)	760,336 (44.6)		2032 (61.7)	762,580 (44.6)	
***Infant sex***				0.108			0.9115			0.921
Male	1,749,365 (51.2)	32,782 (50.9)	1,716,583 (51.2)		5590 (51.2)	1,743,775 (51.2)		2547 (51.1)	1,746,818 (51.2)	
Female	1,668,663 (48.8)	31,661 (49.1)	1,637,002 (48.8)		5323 (48.8)	1,663,340 (48.8)		2437 (48.9)	1,666,226 (48.8)	

^a^ Other races: Asian, American Indian/Alaska Native, Native Hawaiian, Pacific Islander or mixed races; ^b^ PNC: prenatal care; PNC adequate: early and >10 visits; PNC inadequate: late and ≤10 visits.

**Table 3:** Logistic regression analysis for the main effects of chlamydia, gonorrhea, and syphilis (Model 1) and prenatal care (PNC) utilization status (Model 2) on preterm birth and low birthweight.

Variables	Preterm Birth		Low Birthweight	
	aOR	95% CI	aOR	95% CI
Model 1				
Chlamydia	1.02	1.01, 1.04	1.04	1.00, 1.06
No chlamydia (ref.)	1.00		1.00	
Gonorrhea	1.13	1.07, 1.20	1.14	1.07, 1.21
No gonorrhea (ref.)	1.00		1.00	
Syphilis	1.39	1.28, 1.51	1.34	1.22, 1.46
No syphilis (ref.)	1.00		1.00	
Model 2				
PNC adequate	0.36	0.35, 0.36	0.41	0.40, 0.41
PNC inadequate (ref.)	1.00		1.00	

PNC adequate: early and >10 visits; PNC inadequate: late and ≤10 visits; aOR: adjusted odds ratio; CI: confidence interval; Model 1 adjusted for: race/ethnicity, age, education, marital status, health insurance, parity, prior PTB, gestational diabetes, gestational hypertension, smoking, PNC utilization status, and infant sex; Model 2 adjusted for: race/ethnicity, age, education, marital status, health insurance, parity, prior PTB, gestational diabetes, gestational hypertension, smoking, STIs, and infant sex.

**Table 4:** Logistic regression analyses for the interaction effects between chlamydia, gonorrhea, and syphilis (Factor 1) and prenatal care (PNC) utilization status (Factor 2) on preterm birth.

Factor 1	Factor 2	No Preterm Birth	Preterm Birth		*p* Value
		*n*	*n*	aOR (95%CI)	
Chlamydia (−)	PNC (−)	45,127	12,999	3.11 (3.05, 3.18)	<0.001
Chlamydia (−)	PNC (+)	3,034,729	260,730	1.00	
Chlamydia (+)	PNC (−)	1004	384	3.56 (3.15, 4.02)	<0.001
Chlamydia (+)	PNC (+)	56,962	6093	1.02 (0.99, 1.05)	0.229
**Multiplicative Interaction**				1.12 (1.01, 1.25)	0.054
**Relative Excess Risk due to Interaction (RERI)**				0.43 (0.32, 0.53)	<0.001
**Attributable Proportion (AP)**				0.12 (0.02, 0.22)	0.023
**Synergy Index (SI)**				1.20 (1.10, 1.31)	<0.001
Gonorrhea (−)	PNC (−)	45,878	13,232	3.11 (3.05, 3.18)	<0.001
Gonorrhea (−)	PNC (+)	3,082,379	265,626	1.00	
Gonorrhea (+)	PNC (−)	253	151	5.06 (4.10, 6.25)	<0.001
Gonorrhea (+)	PNC (+)	9312	1197	1.12 (1.05, 1.19)	0.000
**Multiplicative Interaction**				1.45 (1.22, 1.72)	0.032
**Relative Excess Risk due to Interaction (RERI)**				1.83 (1.66, 2.00)	<0.001
**Attributable Proportion (AP)**				0.36 (0.19, 0.53)	<0.001
**Synergy Index (SI)**				1.82 (1.65, 1.99)	<0.001
Syphilis (−)	PNC (−)	45,976	13,289	3.12 (3.05, 3.18)	<0.001
Syphilis (−)	PNC (+)	3,087,591	266,188	1.00	
Syphilis (+)	PNC (−)	155	94	5.54 (4.24, 7.24)	<0.001
Syphilis (+)	PNC (+)	4100	635	1.30 (1.20, 1.42)	<0.001
**Multiplicative Interaction**				1.36 (1.10, 1.70)	0.040
**Relative Excess Risk due to Interaction (RERI)**				2.12 (1.90, 2.34)	<0.001
**Attributable Proportion (AP)**				0.38 (0.16, 0.60)	0.000
**Synergy Index (SI)**				1.88 (1.66, 2.09)	<0.001

*n*: number; aOR: adjusted odds ratio; CI: confidence interval; PNC (+): prenatal care adequate; PNC (−): prenatal care inadequate; Adjusted for: Race/ethnicity, age, education, marital status, health insurance, parity, prior preterm birth, gestational diabetes, gestational hypertension, smoking, and infant sex; RERI: Relative Excess Risk due to Interaction; AP: Attributable Proportion; SI: Synergy Index.

**Table 5:** Logistic regression analyses for the interaction effects between chlamydia, gonorrhea, and syphilis (Factor 1) and prenatal care (PNC) utilization status (Factor 2) on low birthweight.

Factor 1	Factor 2	No Low Birthweight	Low Birthweight		*p* Value
		*n*	*n*	aOR (95%CI)	
Chlamydia (−)	PNC (−)	48,511	9615	2.48 (2.42, 2.54)	<0.001
Chlamydia (−)	PNC (+)	3,089,803	205,656	1.00	
Chlamydia (+)	PNC (−)	1084	304	2.76 (2.42, 3.16)	<0.001
Chlamydia (+)	PNC (+)	57,370	5685	1.04 (1.01, 1.07)	0.006
**Multiplicative Interaction**				1.07 (0.95, 1.20)	0.294
**Relative Excess Risk due to Interaction (RERI)**				0.24 (0.12, 0.34)	<0.001
**Attributable Proportion (AP)**				0.09 (−0.03, 0.20)	0.146
**Synergy Index (SI)**				1.16 (1.04, 1.27)	<0.001
Gonorrhea (−)	PNC (−)	49,312	9798	2.47 (2.42, 2.53)	<0.001
Gonorrhea (−)	PNC (+)	3,137,826	210,179	1.00	
Gonorrhea (+)	PNC (−)	283	121	3.64 (2.92, 4.55)	<0.001
Gonorrhea (+)	PNC (+)	9347	1162	1.14 (1.07, 1.21)	<0.001
**Multiplicative Interaction**				1.29 (1.07, 1.56)	0.039
**Relative Excess Risk due to Interaction (RERI)**				1.03 (0.84, 1.22)	<0.001
**Attributable Proportion (AP)**				0.28 (0.09, 0.47)	0.003
**Synergy Index (SI)**				1.64 (1.45, 1.83)	<0.001
Syphilis (−)	PNC (−)	49,409	9856	2.48 (2.42, 2.54)	<0.001
Syphilis (−)	PNC (+)	3,143,011	210,768	1.00	
Syphilis (+)	PNC (−)	186	63	3.22 (2.39, 4.35)	<0.001
Syphilis (+)	PNC (+)	4162	573	1.32 (1.20, 1.44)	<0.001
**Multiplicative Interaction**				0.98 (0.76, 1.28)	0.909
**Relative Excess Risk due to Interaction (RERI)**				0.42 (0.16, 0.68)	<0.001
**Attributable Proportion (AP)**				0.13 (0.11, 0.39)	0.033
**Synergy Index (SI)**				1.23 (0.97, 1.49)	0.055

*n*: number; aOR: adjusted odds ratio; CI: confidence interval; PNC (+): prenatal care adequate; PNC (−): prenatal care inadequate; Adjusted for: race/ethnicity, age, education, marital status, health insurance, parity, prior preterm birth, gestational diabetes, gestational hypertension, smoking, and infant sex.; RERI: Relative Excess Risk due to Interaction; AP: Attributable Proportion; SI: Synergy Index.

## Data Availability

The dataset analyzed for the current study is publicly available from the National Center for Health Statistics (NCHS) Vital Statistics Natality Birth Data and can be accessed through the National Bureau of Economic Research website: https://www.nber.org/research/data/vital-statistics-natality-birth-data (accessed on 15 June 2022).

## Supplementary Materials

The following supporting information can be downloaded at: https://www.mdpi.com/article/10.3390/jcm11175184/s1, Table S1: Logistic regression analyses for the interaction effects between chlamydia, gonorrhea, and syphilis infection (Factor 1) and prenatal care (PNC) utilization status (Factor 2) on preterm birth stratified by maternal age < 25 years, United States, 2019; Table S2: Logistic regression analyses for the interaction effects between chlamydia, gonorrhea, and syphilis infection (Factor 1) and prenatal care (PNC) utilization status (Factor 2) on preterm birth stratified by maternal age ≥ 25, United States, 2019; Table S3: Logistic regression analyses for the interaction effects between chlamydia, gonorrhea, and syphilis (Factor 1) and prenatal care (PNC) utilization status (Factor 2) on low birthweight stratified by maternal age < 25 years, United States; Table S4: Logistic regression analyses for the interaction between chlamydia, gonorrhea, and syphilis infection (Factor 1) and prenatal care (PNC) utilization status (Factor 2) on low birthweight stratified by maternal age ≥ 25, United States; Table S5: Inconsistencies in the interaction analyses on preterm birth before the PNC variable was recoded as a risk factor; Table S6: Inconsistencies in the interaction analyses on low birthweight before the PNC variable as recoded a risk factor.
