# Supplementary material for: Interaction Effects of Maternal Sexually Transmitted Infections with Prenatal Care Utilization Status on Preterm Birth and Low Birthweight: U.S. National Data
Source: J Clin Med. 2022 Sep 1;11(17):5184. doi: 10.3390/jcm11175184 (PMC9456665; doi:10.3390/jcm11175184)
Supplement: Supplementary file 1 [file jcm-11-05184-s001.zip › jcm-1885496-supplementary.pdf]

SUPPLEMENTARY INFORMATION

Interaction Effects of Maternal Sexually Transmitted Infections with Prenatal Care Utilization Status on Preterm Birth and Low Birthweight: US National Data

Sensitivity analysis

Table S1. Logistic regression analyses for the interaction effects between chlamydia, gonorrhea, and syphilis infection (*Factor 1)* and prenatal care (PNC) utilization status (*Factor 2*) on preterm birth stratified by maternal age <25 years

			Maternal age <25		
**Factor 1**	**Factor 2**	**No Preterm Birth n=741,220**	**Preterm Birth n=67,803**		***p* value**
		**n**	**n**	**aOR (95%CI)**	
Chlamydia (-)	PNC (-)	14,936	4,131	3.19 (3.07, 3.30)	<.001
Chlamydia (-)	PNC (+)	690,790	59,950	1.00	
Chlamydia (+)	PNC (-)	573	189	3.29 (2.78, 3.90)	<.001
Chlamydia (+)	PNC (+)	34,921	3,533	1.03 (0.99, 1.07)	0.100
**Multiplicative Interaction**				1.01 (0.86, 1.16)	0.987
**Relative Excess Risk due to Interaction (RERI)**				0.07 (-0.08, 0.22)	0.365
**Attributable Proportion (AP)**				0.02 (-0.13, 0.17)	0.792
**Synergy Index (SI)**				1.03 (0.88, 1.18)	0.690
Gonorrhea (-)	PNC (-)	15,379	4,271	3.18 (3.07, 3.30)	<.001
Gonorrhea (-)	PNC (+)	720,448	62,882	1.00	
Gonorrhea (+)	PNC (-)	130	49	3.58 (2.56, 5.02)	<.001
Gonorrhea (+)	PNC (+)	5,263	601	1.07 (0.98, 1.16)	0.137
**Multiplicative Interaction**				1.05 (0.78, 1.41)	0.762
**Relative Excess Risk due to Interaction (RERI)**				0.33 (0.04, 0.62)	0.027
**Attributable Proportion (AP)**				0.09 (-0.20, 0.38)	0.548
**Synergy Index (SI)**				1.15 (0.85, 1.43)	0.308
Syphilis (-)	PNC (-)	15,454	4,297	3.18 (3.07, 3.30)	<.001
Syphilis (-)	PNC (+)	724,379	63,305	1.00	
Syphilis (+)	PNC (-)	55	23	4.36 (2.66, 7.15)	<.001
Syphilis (+)	PNC (+)	1,332	178	1.25 (1.06, 1.47)	0.006
**Multiplicative Interaction**				1.10 (0.71, 1.69)	0.726
**Relative Excess Risk due to Interaction (RERI)**				0.93 (0.49, 1.36)	<.001
**Attributable Proportion (AP)**				0.21 (-0.22, 0.65)	0.342
**Synergy Index (SI)**				1.38 (0.95, 1.82)	0.050

n: number; aOR: adjusted odds ratio; CI: confidence interval; PNC (+): prenatal care adequate; PNC (-): prenatal care inadequate; Adjusted for: race/ethnicity, education, marital status, health insurance, parity, prior preterm birth, gestational diabetes, gestational hypertension, smoking, and infant sex; RERI: Relative Excess Risk due to Interaction; AP: Attributable Proportion; SI: Synergy Index

Table S2. Logistic regression analyses for the interaction effects between chlamydia, gonorrhea, and syphilis infection (*Factor 1)* and prenatal care (PNC) utilization status (*Factor 2*) on preterm birth stratified by maternal age ≥25

Maternal age ≥25 years old					
**Factor 1**	**Factor 2**	**No Preterm birth n=2,396,602**	**Preterm birth n=212,403**		***p* value**
		**n**	**n**	**aOR (95%CI)**	
Chlamydia (-)	PNC (-)	30,191	8,868	3.05 (2.98, 3.13)	<.001
Chlamydia (-)	PNC (+)	2,343,939	200,780	1.00	
Chlamydia (+)	PNC (-)	431	195	3.59 (3.01, 4.29)	<.001
Chlamydia (+)	PNC (+)	22,041	2,560	0.98 (0.94, 1.02)	0.413
**Multiplicative Interaction**				1.20 (1.03, 1.39)	0.046
**Relative Excess Risk due to Interaction (RERI)**				0.56 (0.41, 0.71)	<.001
**Attributable Proportion (AP)**				0.15 (0.01, 0.30)	0.040
**Synergy Index (SI)**				1.27 (1.12, 1.42)	<.001
Gonorrhea (-)	PNC (-)	30,499	8,961	3.05 (2.97, 3.13)	<.001
Gonorrhea (-)	PNC (+)	2,361,931	202,744	1.00	
Gonorrhea (+)	PNC (-)	123	102	6.15 (4.66, 8.10)	<.001
Gonorrhea (+)	PNC (+)	4,049	596	1.15 (1.05, 1.25)	0.002
**Multiplicative Interaction**				1.75 (1.31, 2.35)	0.034
**Relative Excess Risk due to Interaction (RERI)**				2.95 (2.66, 3.24)	<.001
**Attributable Proportion (AP)**				0.48 (0.19, 0.77)	0.001
**Synergy Index (SI)**				2.34 (2.05, 2.63)	<.001
Syphilis (-)	PNC (-)	30,522	8,992	3.06 (2.98, 3.13)	<.001
Syphilis (-)	PNC (+)	2,363,212	202,883	1.00	
Syphilis (+)	PNC (-)	100	71	6.00 (4.36, 8.26)	<.001
Syphilis (+)	PNC (+)	2,768	457	1.33 (1.20, 1.47)	<.001
**Multiplicative Interaction**				1.47 (0.95, 2.28)	0.253
**Relative Excess Risk due to Interaction (RERI)**				2.61 (2.17, 3.04)	<.001
**Attributable Proportion (AP)**				0.43 (0.00, 0.87)	0.049
**Synergy Index (SI)**				2.09 (1.66, 2.53)	<.001

n: number; aOR: adjusted odds ratio; CI: confidence interval; PNC (+): prenatal care adequate; PNC (-): prenatal care inadequate; Adjusted for: race/ethnicity, education, marital status, health insurance, parity, prior preterm birth, gestational diabetes, gestational hypertension, smoking, and infant sex; RERI: Relative Excess Risk due to Interaction; AP: Attributable Proportion; SI: Synergy Index

Table S3. Logistic regression analyses for the interaction effects between chlamydia, gonorrhea, and syphilis (*Factor 1*) and prenatal care (PNC) utilization status (*Factor 2*) on low birthweight stratified by maternal age <25 years

Maternal age <25					
**Factor 1**	**Factor 2**	**No low birthweight n=749,038**	**Low birthweight n=59,985**		***p* value**
		**n**	**n**	**aOR (95%CI)**	
Chlamydia (-)	PNC (-)	15,977	3,090	2.38 (2.29, 2.48)	<.001
Chlamydia (-)	PNC (+)	697,524	53,216	1.00	
Chlamydia (+)	PNC (-)	614	148	2.49 (2.07, 3.00)	<.001
Chlamydia (+)	PNC (+)	34,923	3,531	1.06 (1.02, 1.10)	0.001
**Multiplicative Interaction**				0.99 (0.83, 1.17)	0.886
**Relative Excess Risk due to Interaction (RERI)**				0.05 (-0.11, 0.22)	0.572
**Attributable Proportion (AP)**				0.02 (-0.15, 0.19)	0.826
**Synergy Index (SI)**				1.03 (0.87, 1.20)	0.696
Gonorrhea (-)	PNC (-)	16,454	3,196	2.38 (2.28, 2.48)	<.001
Gonorrhea (-)	PNC (+)	727,237	56,093	1.00	
Gonorrhea (+)	PNC (-)	137	42	2.85 (2.00, 4.07)	<.001
Gonorrhea (+)	PNC (+)	5,210	654	1.14 (1.05, 1.24)	0.001
**Multiplicative Interaction**				1.05 (0.77, 1.44)	0.786
**Relative Excess Risk due to Interaction (RERI)**				0.33 (0.02, 0.64)	0.039
**Attributable Proportion (AP)**				0.11 (-0.20, 0.43)	0.479
**Synergy Index (SI)**				1.29 (0.98, 1.61)	0.051
Syphilis (-)	PNC (-)	16,532	3,219	2.38 (2.28, 2.47)	<.001
Syphilis (-)	PNC (+)	731,123	56,561	1.00	
Syphilis (+)	PNC (-)	59	19	3.43 (2.02, 5.82)	<.001
Syphilis (+)	PNC (+)	1,324	186	1.35 (1.15, 1.57)	<.001
**Multiplicative Interaction**				1.07 (0.66, 1.71)	0.818
**Relative Excess Risk due to Interaction (RERI)**				0.70 (0.23, 1.17)	0.003
**Attributable Proportion (AP)**				0.20 (-0.27, 0.68)	0.405
**Synergy Index (SI)**				1.40 (0.93, 1.88)	0.057

n: number; aOR: adjusted odds ratio; CI: confidence interval; PNC (+): prenatal care adequate; PNC (-): prenatal care inadequate; Adjusted for: race/ethnicity, education, marital status, health insurance, parity, prior preterm birth, gestational diabetes, gestational hypertension, smoking, and infant sex; RERI: Relative Excess Risk due to Interaction; AP: Attributable Proportion; SI: Synergy Index

Table S4. Logistic regression analyses for the interaction between chlamydia, gonorrhea, and syphilis infection (*Factor 1*) and prenatal care (PNC) utilization status (*Factor 2*) on low birthweight stratified by maternal age ≥25

Maternal age ≥25 years old					
**Factor 1**	**Factor 2**	**No Low birthweight n=2,447,730**	**Low birthweight n=161,275**		***p* value**
		**n**	**n**	**aOR (95%CI)**	
Chlamydia (-)	PNC (-)	32,534	6,525	2.50 (2.43, 2.57)	<.001
Chlamydia (-)	PNC (+)	2,392,279	152,440	1.00	
Chlamydia (+)	PNC (-)	470	156	2.83 (2.34, 3.42)	<.001
Chlamydia (+)	PNC (+)	22,447	2,154	0.98 (0.94, 1.03)	0.382
**Multiplicative Interaction**				1.05 (0.89, 1.23)	0.606
**Relative Excess Risk due to Interaction (RERI)**				0.35 (0.18, 0.51)	<.001
**Attributable Proportion (AP)**				0.12 (-0.04, 0.29)	0.416
**Synergy Index (SI)**				1.24 (1.07, 1.40)	0.001
Gonorrhea (-)	PNC (-)	32,858	6,602	2.49 (2.42, 2.57)	<.001
Gonorrhea (-)	PNC (+)	2,410,589	154,086	1.00	
Gonorrhea (+)	PNC (-)	146	79	4.09 (3.07, 5.47)	<.001
Gonorrhea (+)	PNC (+)	4,137	508	1.08 (0.99, 1.19)	0.094
**Multiplicative Interaction**				1.52 (1.20, 1.93)	0.024
**Relative Excess Risk due to Interaction (RERI)**				1.52 (1.28, 1.76)	<.001
**Attributable Proportion (AP)**				0.37 (0.13, 0.61)	0.002
**Synergy Index (SI)**				1.97 (1.07, 1.40)	<.001
Syphilis (-)	PNC (-)	32,877	6,637	2.50 (2.43, 2.57)	<.001
Syphilis (-)	PNC (+)	2,411,888	154,207	1.00	
Syphilis (+)	PNC (-)	127	44	3.03 (2.10, 4.35)	<.001
Syphilis (+)	PNC (+)	2,838	387	1.30 (1.16, 1.45)	<.001
**Multiplicative Interaction**				0.93 (0.68, 1.27)	0.655
**Relative Excess Risk due to Interaction (RERI)**				0.23 (-0.08, 0.54)	0.150
**Attributable Proportion (AP)**				0.07 (-0.24, 0.39)	0.647
**Synergy Index (SI)**				1.13 (0.81, 1.44)	0.415

n: number; aOR: adjusted odds ratio; CI: confidence interval; PNC (+): prenatal care adequate; PNC (-): prenatal care inadequate; Adjusted for: race/ethnicity, education, marital status, health insurance, parity, prior preterm birth, gestational diabetes,

gestational hypertension, smoking, and infant sex; RERI: Relative Excess Risk due to Interaction; AP: Attributable Proportion; SI: Synergy Index

Table S5. Inconsistencies in the interaction analyses on preterm birth before the PNC variable was recoded as a risk factor

Factor 1	Factor 2	No Preterm birth	Preterm birth		*p* value
		n	n	aOR (95%CI)	
**Chlamydia (-)**	**PNC (-)**	**45,127**	**12,999**	**1.00**	
Chlamydia (-)	PNC (+)	3,034,729	260,730	0.32 (0.31, 0.33)	<.001
Chlamydia (+)	PNC (-)	1,004	384	1.14 (1.01, 1.29)	0.0356
Chlamydia (+)	PNC (+)	56,962	6,093	0.33 (0.32, 0.34)	<.001
**Multiplicative Interaction**				**0.92 (0.83, 1.02)**	0.2174
**Relative Excess Risk due to Interaction (RERI)**				**-0.13 (-0.23, -0.02)**	0.0151
**Attributable Proportion (AP)**				**-0.39 (-0.49, -0.28)**	<.001
**Synergy Index (SI)**				**1.24 (1.13, 1.34)**	<.001
Gonorrhea (-)	PNC (-)	45,878	13,232	1.00	
Gonorrhea (-)	PNC (+)	3,082,379	265,626	0.32 (0.31, 0.33)	<.001
Gonorrhea (+)	PNC (-)	253	151	1.63 (1.32, 2.01)	<.001
Gonorrhea (+)	PNC (+)	9,312	1,197	0.36 (0.34, 0.38)	<.001
**Multiplicative Interaction**				**0.69 (0.58, 0.82)**	<.001
**Relative Excess Risk due to Interaction (RERI)**				**-0.59 (-0.76, -0.41)**	<.001
**Attributable Proportion (AP)**				**-1.64 (-1.81, -1.47)**	<.001
**Synergy Index (SI)**				**12.80 (12.60, 13.10)**	<.001
Syphilis (-)	PNC (-)	45,976	13,289	1.00	
Syphilis (-)	PNC (+)	3,087,591	266,188	0.32 (0.32, 0.33)	<.001
Syphilis (+)	PNC (-)	155	94	1.78 (1.36, 2.32)	<.001
Syphilis (+)	PNC (+)	4,100	635	0.42 (0.38, 0.46)	<.001
**Multiplicative Interaction**				**0.79 (0.64, 0.98)**	0.0298
**Relative Excess Risk due to Interaction (RERI)**				**-0.68 (-0.90, -0.46)**	<.001
**Attributable Proportion (AP)**				**-1.62 (-1.84, -1.40)**	<.001
**Synergy Index (SI)**				**-5.80 (-6.00, -5.60)**	<.001

n: number; aOR: adjusted odds ratio; CI: confidence interval; PNC (+): prenatal care adequate; PNC (-): prenatal care inadequate; Adjusted for: race/ethnicity, age, education, marital status, health insurance, parity, prior preterm birth, gestational diabetes, gestational hypertension, smoking, and infant sex; RERI: Relative Excess

Risk due to Interaction; AP: Attributable Proportion; SI: Synergy Index ; **inconsistencies marked in bold**

Table S6. Inconsistencies in the interaction analyses on low birthweight before the PNC variable as recoded a risk factor

Factor 1	Factor 2	No Low birthweight	Low birthweight		*p* value
		n	n	aOR (95%CI)	
**Chlamydia (-)**	**PNC (-)**	**48,511**	**9,615**	**1.00**	
Chlamydia (-)	PNC (+)	3,089,803	205,656	0.40 (0.39, 0.41)	<.001
Chlamydia (+)	PNC (-)	1,084	304	1.12 (0.97, 1.28)	0.112
Chlamydia (+)	PNC (+)	57,370	5,685	0.42 (0.40, 0.43)	<.001
**Multiplicative Interaction**				**0.94 (0.82, 1.05)**	0.331
**Relative Excess Risk due to Interaction (RERI)**				**-0.10 (-0.21, 0.01)**	0.074
**Attributable Proportion (AP)**				**-0.24 (-0.35, -0.12)**	<.001
**Synergy Index (SI)**				**1.21 (1.09, 1.32)**	<.001
Gonorrhea (-)	PNC (-)	49,312	9,798	1.00	
Gonorrhea (-)	PNC (+)	3,137,826	210,179	0.40 (0.39, 0.41)	<.001
Gonorrhea (+)	PNC (-)	283	121	1.47 (1.18, 1.84)	<.001
Gonorrhea (+)	PNC (+)	9,347	1,162	0.46 (0.43, 0.49)	<.001
**Multiplicative Interaction**				**0.78 (0.59, 0.97)**	0.049
**Relative Excess Risk due to Interaction (RERI)**				**-0.41 (-0.60, -0.22)**	<.001
**Attributable Proportion (AP)**				**-0.89 (-1.08, -0.70)**	<.001
**Synergy Index (SI)**				**4.15 (3.96, 4.34)**	<.001
Syphilis (-)	PNC (-)	49,409	9856	1.00	
Syphilis (-)	PNC (+)	3,143,011	210,768	0.40 (0.39, 0.41)	<.001
Syphilis (+)	PNC (-)	186	63	1.30 (0.96, 1.76)	0.088
Syphilis (+)	PNC (+)	4,162	573	0.53 (0.48, 0.58)	<.001
**Multiplicative Interaction**				**1.02 (0.76, 1.28)**	0.890
**Relative Excess Risk due to Interaction (RERI)**				**-0.17 (-0.43, 0.09)**	0.201
**Attributable Proportion (AP)**				**-0.32 (-0.58, -0.06)**	0.015
**Synergy Index (SI)**				**1.57 (1.30, 1.83)**	<.001

n: number; aOR: adjusted odds ratio; CI: confidence interval; PNC (+): prenatal care adequate; PNC (-): prenatal care inadequate; Adjusted for: race/ethnicity, age,

education, marital status, health insurance, parity, prior preterm birth, gestational diabetes, gestational hypertension, smoking, and infant sex; **inconsistencies marked in bold**
